# Time course of fluid responsiveness in sepsis: the fluid challenge revisiting (FCREV) study

**DOI:** 10.1186/s13054-019-2448-z

**Published:** 2019-05-16

**Authors:** Claire Roger, Laurent Zieleskiewicz, Christophe Demattei, Karim Lakhal, Gael Piton, Benjamin Louart, Jean-Michel Constantin, Russell Chabanne, Jean-Sébastien Faure, Yazine Mahjoub, Isabelle Desmeulles, Hervé Quintard, Jean-Yves Lefrant, Laurent Muller, AzuRea Group, Claire Roger, Laurent Zieleskiewicz, Karim Lakhal, Benjamin Louart, Jean-Michel Constantin, Russell Chabanne, Jean-Sébastien Faure, Yazine Mahjoub, Hervé Quintard, Jean-Yves Lefrant, Laurent Muller

**Affiliations:** 1 Department of Anesthesiology and Intensive Care, Pain and Emergency Medicine, Nîmes-Caremeau University Hospital, Univ Montpellier, Place du Professeur Robert Debré, 30 029 Nîmes Cedex 9, France; 2 Physiology Department. EA 2992, Faculty of Medicine, Univ Montpellier, Montpellier-Nimes University, Nîmes, France; 3 Department of Anesthesiology and Intensive Care Medicine, University Hospital of Marseille, 13000 Marseille, France; 4 Department of Biostatistics Epidemiology and Medical information, Nîmes-Caremeau University Hospital, Univ Montpellier, Place du Professeur Robert Debré, 30 029 Nîmes Cedex 9, France; 5 Department of Anesthesiology and Intensive Care Medicine, University Hospital of Nantes, 44000 Nantes, France; 6 Medical Intensive Care unit, University Hospital of Besançon, 25030 Besançon, France; 7 Department of Anesthesiology and Intensive Care Medicine, University Hospital of Clermont-Ferrand, 63000 Clermont-Ferrand, France; 8 Department of Anesthesiology and Intensive Care Medicine, University Hospital of Amiens, 80000 Amiens, France; 9 Department of Anesthesiology and Intensive Care Medicine, University Hospital of Caen, 14033 Caen, France; 10 Department of Anesthesiology and Intensive Care Medicine, University Hospital of Nice, 06000 Nice, France; 11 Aix Marseille University, INSERM1263, INRA1260, C2VN, Marseille, France

**Keywords:** Fluid responsiveness, Fluid challenge, ICU, Shock, Echocardiography

## Abstract

**Background:**

Fluid challenge (FC) is one of the most common practices in Intensive Care Unit (ICU). The present study aimed to evaluate whether echocardiographic assessment of the response to FC at the end of the infusion or 20 min later could affect the results of the FC.

**Methods:**

This is a prospective, observational, multicenter study including all ICU patients in septic shock requiring a FC of 500 mL crystalloids over 10 min. Fluid responsiveness was defined as a > 15% increase in stroke volume (SV) assessed by velocity-time integral (VTI) measurements at baseline (*T*_0_), at the end of FC (T_10_), then 10 (T_20_) and 20 min (T_30_) after the end of FC.

**Results:**

From May 20, 2014, to January 7, 2016, a total of 143 patients were enrolled in 11 French ICUs (mean age 64 ± 14 years, median IGS II 53 [43–63], median SOFA score 10 [8–12]). Among the 76/143 (53%) patient responders to FC at *T*_10_, 37 patients were transient responders (TR), i.e., became non-responders (NR) at *T*_30_ (49%, 95%CI = [37–60]), and 39 (51%, 95%CI = [38–62]) patients were persistent responders (PR), i.e., remained responders at *T*_30_. Among the 67 NR at *T*_10_, 4 became responders at T30, (6%, 95%CI = [1.9–15.3]). In the subgroup analysis, no statistical difference in hemodynamic and echocardiographic parameters was found between groups.

**Conclusions:**

This study shows that 51.3% of initial responders have a persistent response to fluid 30 min after the beginning of fluid infusion and only 41.3% have a transient response highlighting that fluid responsiveness is time dependent.

**Trial registration:**

ClinicalTrials.gov, NCT02116413. Registered on April 16, 2014

**Electronic supplementary material:**

The online version of this article (10.1186/s13054-019-2448-z) contains supplementary material, which is available to authorized users.

## Background

Fluid therapy is the primary resuscitation maneuver of acute circulatory failure management in critically ill patients [1]. Adequate fluid resuscitation is a key issue as both hypovolemia and fluid overload are associated with poor outcome in intensive care unit (ICU) [2–4]. Despite consistent data published over the last decades, the criteria indicating fluid administration remains highly debated [5–7]. Recent large observational studies have shown that ICU physicians mainly use arterial pressure and heart rate to assess blood volume status, while measurement of cardiac output (CO) or stroke volume (SV) is rarely performed [8, 9]. Even so, the goal of fluid infusion is to increase SV or CO when hypovolemia or preload dependency is suspected. As CO (or its surrogates) better describes blood volume variations than arterial pressure and heart rate [1, 10], international guidelines recommend measuring SV or CO to evaluate fluid status in patients that are not responding to initial resuscitation based on clinical assessment [1, 10]. A positive response to fluid therapy (fluid responsiveness) is defined as a 10–15% SV or CO increase immediately after 250 to 500 ml of fluid infusion [1, 11]. In ICU, transthoracic Doppler echocardiography (TTE) provides a non-invasive estimation of SV by measuring the velocity time integral (VTI) of sub-aortic blood flow [1, 7, 12]. A 15% VTI increase is used to define fluid responders [1, 12, 13].

Beyond immediate response to fluid infusion, the efficacy of a fluid bolus over time is affected by various parameters such as blood volume status, cardiac function, type of infused fluid, and capillary leak severity [14]. Little data is available to describe the time course of fluid efficacy defined as a greater than 15% CO increase. It could be hypothesized that, after a fluid bolus, the initial SV or CO increase could not be sustained over time. Therefore, we could imagine that a patient initially identified as a fluid responder could no longer be responder 30 min after fluid infusion, leading to discrepancies in fluid management decision-making.

The primary aim of the present study was to determine the proportion of patients changing from a responder status immediately after a 10-min fluid infusion to a non-responder status 20 min after the end of infusion.

## Methods

We conducted a prospective multicenter study involving 11 intensive care units (ICUs) in 8 University Hospitals (Nimes, Marseille, Nice, Clermont-Ferrand, Nantes, Caen, Amiens, Besancon). The protocol was approved by the Nimes University Hospital local ethics committee (Comité de Protection des Personnes #2014.02.06, #ID_RCB 2013-A01702-43) and registered in ClinicalTrials.gov (NCT02116413). Written consent prior to enrolment or in permitted instances, delayed participant or legal surrogate written consent following enrolment was obtained.

### Participants

Adult ICU patients meeting criteria for septic shock and requiring a fluid challenge according to the treating physician were eligible for inclusion.

The inclusion criteria were:

- Patient ≥ 18 year old who gave his (her) informed consent (or his (her) relative) or delayed
- Patient with septic shock according to the Surviving Sepsis Campaign criteria [15]
- Patient on mechanical ventilation without spontaneous breathing
- Patients in whom a fluid challenge was required for the following reasons, according to physician decision:
  - Persistent hypotension despite fluid resuscitation of 20 to 40 mL/kg and requiring vasopressors as indicated in 2012 Surviving Sepsis Campaign criteria [15]
  - ScVO_2_ < 70% or SvO_2_ < 65%.
  - Urine output < 0.5 mL h^−1^ during two consecutive hours
  - Skin mottles
  - Arterial lactate concentration > 2 mMol L^−1^

Non-inclusion criteria were:

- Informed consent not obtained
- Prisoner
- Patient under legal guardianship
- Parturient
- Severe mitral or aortic regurgitation
- Patients with cardiac arrhythmia
- Poor echogenicity

Exclusion criteria were patients in whom a new fluid challenge was required or requiring an increase in vasopressor infusion rate before the end of the protocol.

### Study protocol

#### Intervention, fluid responsiveness definition (Fig. 1)

Included patients were monitored with invasive arterial pressure. The fluid challenge was performed with 500-ml crystalloids (0.9% NaCl or lactated Ringer’s) over 10 min at a constant infusion rate (50 ml min^−1^). Fluid responsiveness was defined as a > 15% increase in SV [1].

**Fig. 1:**
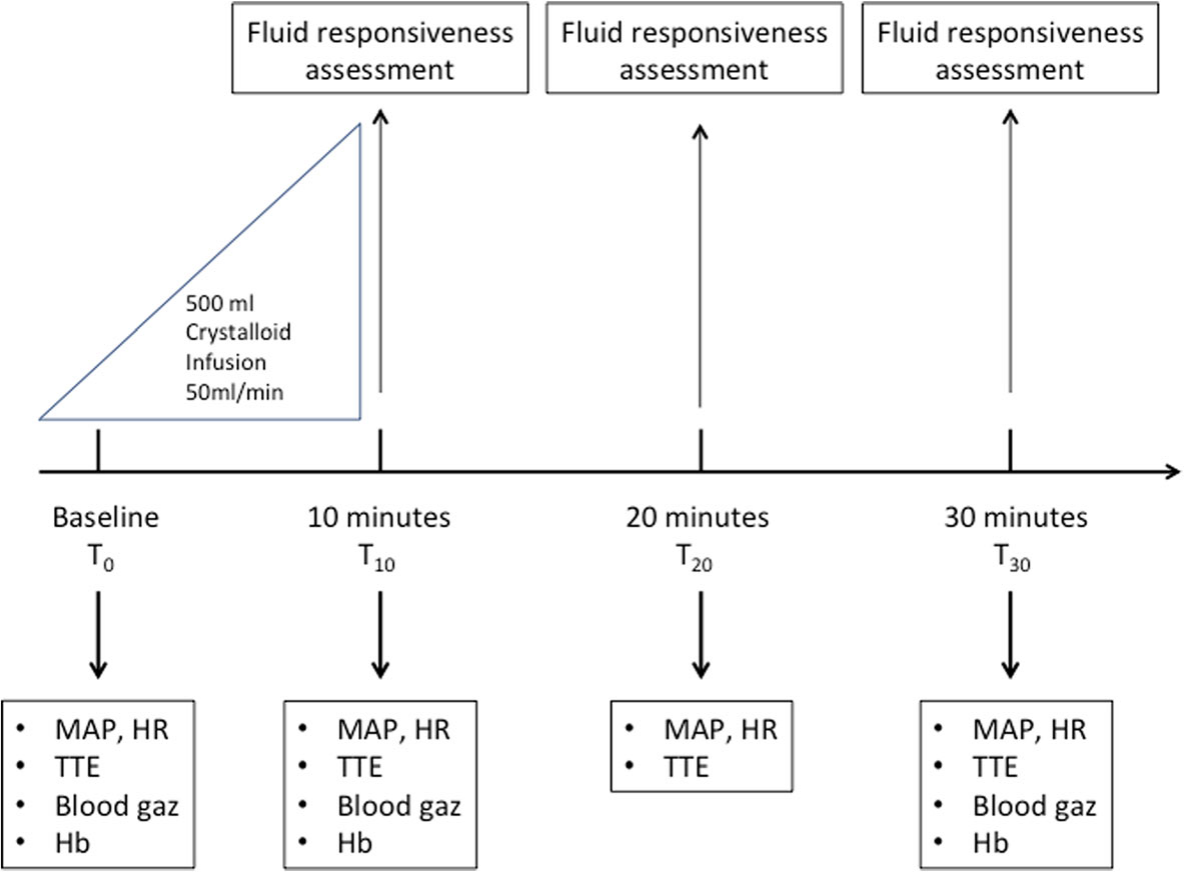
Study design. MAP, mean arterial pressure (mmHg); HR, heart rate (beat per minute), TTE, transthoracic echocardiography; Hb, patient blood hemoglobin (g/dL)

#### Measured parameters and time of measurement

The different studied parameters were collected from baseline (*T*_0_) just before the initiation of fluid challenge until 20 min (T_30_) after the end of fluid challenge. This timing was chosen as fluid challenge is classically performed in 10 to 30 min [11, 16]. Exploring the effect of fluid challenge after *T*_30_ was considered as non-ethical as it potentially delayed a new fluid challenge.

Patient characteristic, age, sex, height, weight, main diagnosis, and the New Simplified Acute Physiology Score II at admission, were collected.

The ventilator settings (tidal volume (VT), respiratory rate (RR), FiO2, positive-end expiratory pressure (PEEP), and plateau pressure (Pplat)) and the Score of Organ Failure Assessment (SOFA) were collected at patient inclusion and were not modified during study intervention.

Invasive mean arterial pressure (MAP) and heart rate (HR) were continuously monitored.

Transthoracic echocardiography (TTE) assessment was performed by experienced operator. Patients were preferentially assessed in semi-recumbent position that was not altered during the experiment. The cardiac function was assessed on a five chamber apical view. Stroke volume and its variations during the experiment were assessed by maximal VTI and its variations over time [1]. The maximal subaortic VTI was recorded independently of the respiratory cycle. Left ventricular filling pressures and diastolic function were assessed by recording transmitral pulsed Doppler diastolic inflow (E wave velocity (cm s^−1^), A wave velocity (cm s^−1^), E/A velocity ratio) coupled to tissue Doppler imaging at the lateral mitral annulus (Ea wave velocity (cm s^−1^)). For right ventricular function assessment, the right to left surface telediastolic ventricular ratio and the tissue Doppler imaging at the lateral tricuspid annulus (tricuspid S wave velocity (cm s^−1^)) were measured [17, 18]. MAP, HR, and TTE variables were collected at baseline (*T*_0_), at the end of fluid challenge (*T*_10_), and 10 (*T*_20_) and 20 min (*T*_30_) after the end of fluid challenge.

Blood samples were withdrawn at *T*_0_, *T*_10_, and *T*_30_ for measuring P_a_O_2_, P_a_CO_2_, pH, S_a_O_2_, *central venous oxygen saturation* (S_cv_O_2_), arterial lactate (m mol^−1^) and hemoglobin (g dl^−1^) concentrations.

### Statistical analysis

The primary and secondary analyses were performed according to the intention-to-treat principle. A per-protocol analysis was also performed for the primary criteria by excluding patients for whom the fluid challenge protocol was not respected.

The primary endpoint was the rate of NR to fluid challenge at *T*_30_ among the responders at *T*_10_ (fluid responsiveness was defined by a > 15% VTI increase, corresponding to a > 15% SV increase). This rate was estimated with its 95% confidence interval and compared to a fixed proportion of 10% with a unilateral risk alpha.

We calculated that 69 responders to fluid challenge at *T*_10_ were necessary to have a power of 80% to detect a rate of non-responders at *T*_30_ greater than 10%, with a unilateral alpha risk of 5%. Under the hypothesis that 50% of patients would be responders at *T*_10_, the inclusion of 138 patients was judged necessary. For taking a 5% rate of unusable data into account, 145 patients were included.

Quantitative data are expressed as mean and standard deviation (SD) or median and interquartile range (IQR), according to their distribution. Qualitative data are expressed as absolute number and frequency (%).

Comparison between groups used, when appropriate, ANOVA or Kruskal-Wallis test for three group comparisons and student’s *T*, Wilcoxon, chi-square, or Fisher’s tests for two group comparisons. When multiple comparisons were performed, a Bonferroni correction was applied. A *p* value < 0.05 was considered as statistically significant. Statistical analysis was performed using R software version 3.0.2 (R Development Core Team 2009, R Foundation for Statistical Computing, Vienna, Austria).

## Results

### Patient characteristics and flow chart

**Fig. 2:**
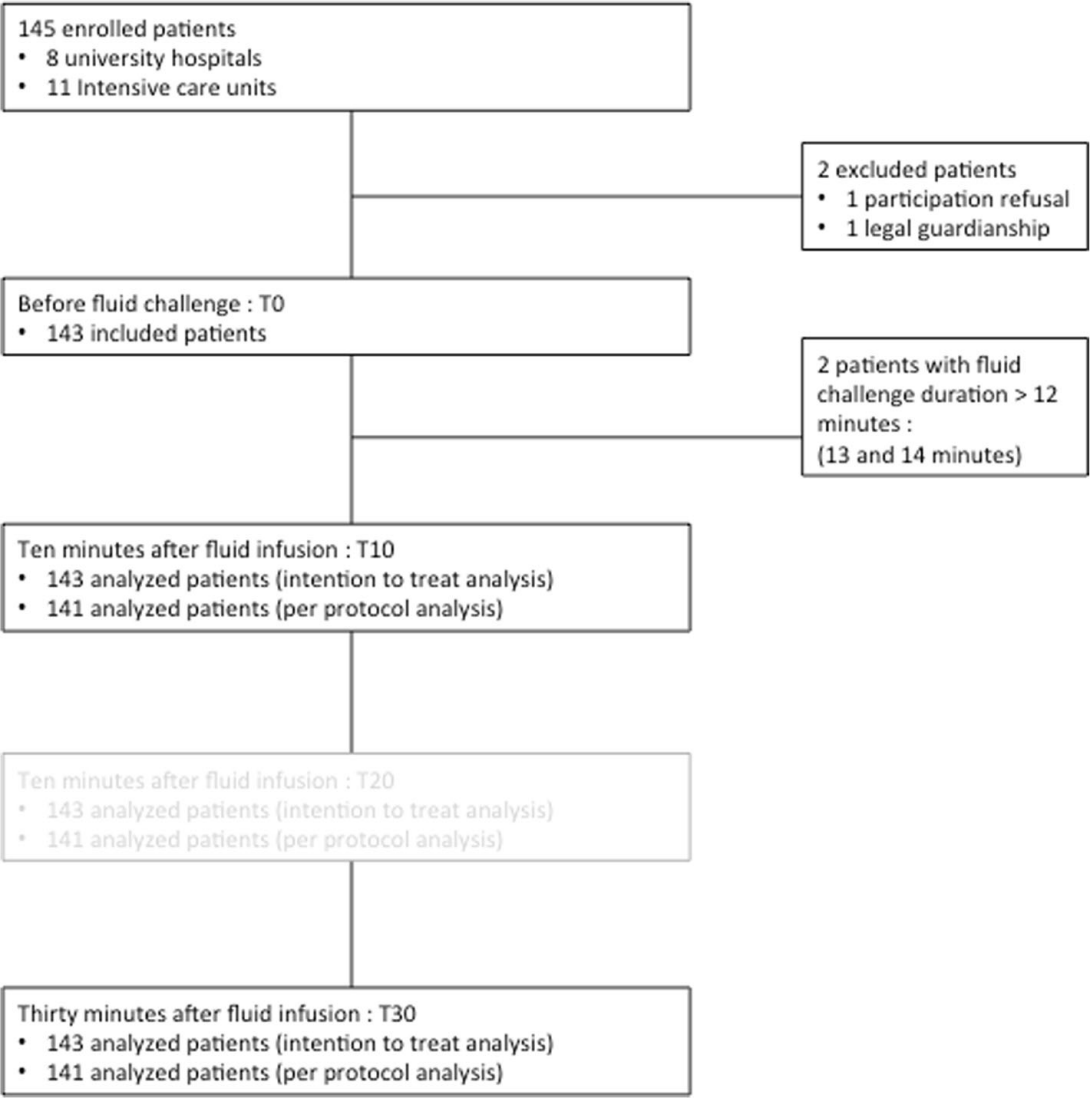
Study flow chart

From May 20, 2014, to January 7, 2016, 143 out of 145 patients eligible with septic shock were included (Fig. 2). All patients received norepinephrine. Patient characteristics are shown in Table 1. Patient cardio-respiratory, biological, and echocardiographic parameters are shown in Table 2.

**Table 1:** Patient characteristics (*n* = 143)

Variables	
Female	50 (35%)
Age (years)	64 ± 14
Weight (kg)	75 ± 20
Height (cm)	168 ± 9
BMI (kg m^−2^)	26.8 ± 7.1
SAPS II score at admission	53 [43–63]
SOFA score at inclusion	10 [8–12]
*Comorbidities (n, %)*	
Hypertension	53 (37%)
Coronary artery disease	24 (17%)
Cardiac heart failure	5 (4%)
Diabetes mellitus	17 (12%)
COPD	17 (12%)
Creatinine clearance < 30 ml min^−1^	8 (6%)
Origin of sepsis (*n*, %)	
Pulmonary	62 (43%)
Intra-abdominal	58 (41%)
Urinary tract	12 (8%)
Bacteremia	7 (5%)
Miscellaneous	15 (11%)

Continuous data are presented as the mean (SD) or median (IQR). Categorical data are presented as counts (%)

*BMI* body mass index, *SAPS II* Simplified Acute Physiology Score II, *SOFA* Sequential Organ Failure Assessment, *COPD* chronic obstructive pulmonary disease

**Table 2:** Patient clinical, biological, and echocardiographic parameters at baseline (*n* = 143)

Variables	
Heart rate (bpm) (MD = 0)	94 ± 24
Mean arterial pressure (mmHg) (MD = 0)	74 ± 12
Tidal volume (mL/Kg) (MD = 0)	6.0 ± 1.0
Respiratory rate (cycle min^−1^) (MD = 0)	22 [18–25]
PEEP (cm H_2_O) (MD = 0)	6 [5–8]
*P*_plateau_ (cm H_2_O) (MD = 9)	19 [17–23]
Norepinephrine infusion rate (μg kg min^−1^)	0.41 [0.22–0.95]
Arterial blood lactate (mMol L^−1^) (MD = 5)	1.95 [1.3–3.55]
S_cv_O_2_ (MD = 58)	77 [68–83]
pH (MD = 2)	7.34 [7.25–7.40]
P_a_O_2_ (mmHg) (MD = 2)	99 [79–128]
P_a_CO_2_ (mmHg) (MD = 3)	40 [35–46]
Hemoglobin (g dL^−1^) (MD = 2)	10.7 [9.4–12.1]
Estimated ejection fraction (MD = 7)	
< 40%	16 (11%)
≥ 40%	120 (84%)
VTI (cm) (MD = 0)	16.6 ± 5
E wave (m s^−1^) (MD = 1)	0.69 ± 0.20
A wave (m s^−1^) (MD = 3)	0.74 ± 0.23
E′ wave (m s^−1^) (MD = 2)	0.1 [0.07–0.13]
S wave (cm s^−1^) (MD = 13)	11.7 ± 5.1
E/A ratio (MD = 3)	1.03 ± 0.55
E/E′ ratio (MD = 3)	7.0 ± 3.7

Continuous data are presented as the mean (SD) or median (IQR). Categorical data are presented as counts (%)

*MD* missing data, *S*_*cv*_*O*_*2*_ central venous oxygen saturation

#### Fluid challenge assessment

**Fig. 3:**
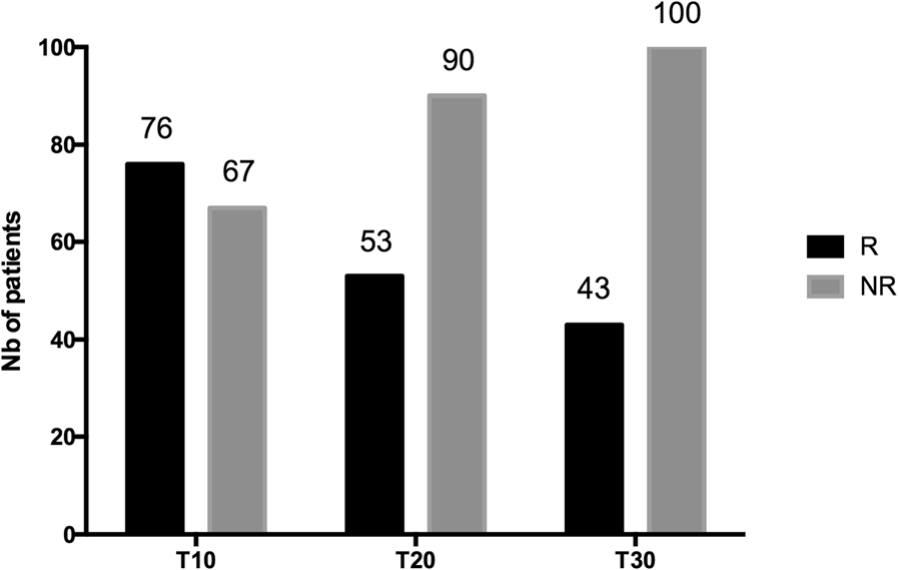
Distribution of responders and non-responders to fluid challenge over time, according to velocity time integral (VTI) measurement at T10, T20 and T30

**Fig. 4:**
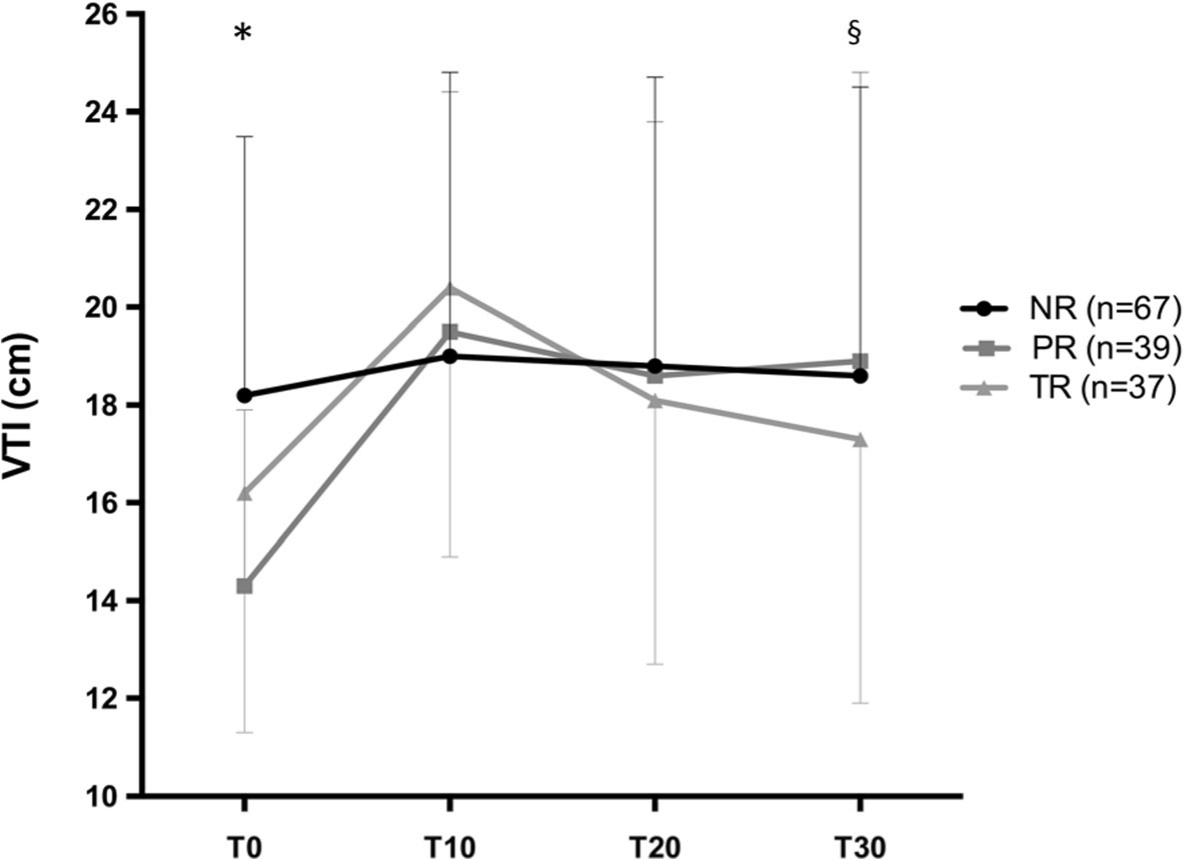
Velocity time integral (VTI) course over time in non-responders (NR), transient responders (TR), and persistent responders (PR). *Difference between VTI value at baseline between NR, TR, and NR groups (*p* = 0.0003). ^§^Significant interaction between time and groups (*p* < 0.0001): stability of VTI over time in the NR group, VTI increase from *T*_0_ to *T*_10_, and subsequent decrease from T10 to T30 in TR and PR groups, with crossing curves between T_10_ and T_20_

Fluid challenge induced a > 15% increase in VTI in 76/143 patients (53%) at *T*_10_ (Fig. 3). Among these 76 responders, 37 changed their fluid responsiveness status from R to NR at *T*_30_ (48.7%, 95%CI = [37.2–60.3]). These patients were defined as transient responders (TR). Among the 76 responders, 39 (51.3%, 95%CI = [39.6–62.8]) remained responders (R) at *T*_30._ These patients were defined as persistent responders (PR) (Fig. 3). Among 67 NR at *T*_10_, only 4 became R at *T*_30_ (6%, 95%CI = [1.9–15.3]). The per-protocol analysis was achieved on 141 patients, and results are comparable to the results of the intention-to-treat analysis presented above (percentage estimations do not differ more than 0.6%). Hemodynamic and echocardiographic time profile of NR, TR, and PR are shown in Tables 3 and 4 (Additional file 1). The time course of VTI in NR, TR, and PR groups is shown in Fig. 4.

**Table 3:** Comparison of patient characteristics between non-responders (NR), persistent responders (PR), and transient responders (TR)

Variables	NR (*n* = 67)	PR (*n* = 39)	TR (*n* = 37)	*p* value for all groups	*p* value NR vs. PR	*p* value NR vs. TR	*p* value PR vs. TR
Age (years)	62.6 ± 13.7	67.7 ± 14.1	64.5 ± 14.1	0.19	0.07	0.50	0.33
SAPS II at admission	48.5 [42.3–57.8]	58 [48–73.5]	55 [45–62]	0.053	0.017	0.23	0.29
SOFA at inclusion	9 [8–11.8]	10 [9–13]	10 [7–11]	0.34	0.14	0.86	0.33
Arterial lactate at baseline (mMol/l)	1.8 [1.3–3.1]	2.55 [1.33–5.43]	1.9 [1.1–3.45]	0.16	0.10	0.78	0.08
ICU mortality rate	25.4%	46.2%	37.8%	0.08	0.048	0.27	0.62
Norepinephrine ≥ 0.5 μg kg^−1^ min^−1^	0.34 [0.22–0.67]	0.64 [0.32–1.29]	0.39 [0.20–0.95]	0.07	0.02	0.84	0.13
LVEF				0.88	1.00	0.76	0.73
< 40%	7 (11.1%)	4 (10.5%)	5 (14.3%)				
≥ 40%	56 (88.9%)	34 (89.5%)	30 (85.7%)				

Continuous data are presented as the mean (SD) or median (IQR). Categorical data are presented as counts (%). For comparison of the NR, TR, and PR groups, a *p* value < 0.05 was considered as significant. For pairwise comparison, a *p* value < 0.017 was considered as significant (Bonferroni correction)

*SAPS II* Simplified Acute Physiology Score II, *SOFA* Sequential Organ Failure Assessment, *ICU* intensive care unit, *LVEF* left ventricular ejection fraction

**Table 4:** Time-course of hemodynamic and echocardiographic variables according to fluid responsiveness status (non-responders (NR) (*n* = 67), persistent responders (PR) (*n* = 39), and transient responders (TR) (*n* = 37))

Variables		T_0_ (baseline)	T_10_	T_20_	T_30_
MAP (mmHg)	NR	73 ± 9.9	77.7 ± 11.3	76.5 ± 11.1	76 ± 11.7
	PR	75 ± 15.2	81.7 ± 15.6	82.6 ± 14.8	80.8 ± 14.6
	TR	73.1 ± 10.7	81.4 ± 13.2	76.7 ± 14.4	75.9 ± 12.6
SAP (mmHg)	NR	114.7 ± 19.5	123.8 ± 22.2	122.4 ± 21.5	120.3 ± 21.7
	PR	112.2 ± 22.8	126.2 ± 22.4	125 ± 21.4	121.3 ± 22
	TR	111.9 ± 18.5	127.3 ± 21.6	120.4 ± 21.3	118.9 ± 20.8
DAP (mmHg)	NR	54.6 ± 8.9	57.3 ± 9.8	56.4 ± 9.8	56.1 ± 9.8
	PR	59.4 ± 13	62.1 ± 12.2	61.6 ± 13.1	60.7 ± 12.2
	TR	55.2 ± 8.8	59 ± 10.7	56.6 ± 11.5	56.5 ± 10.7
HR (bpm)	NR	93.5 ± 22.1	91.3 ± 21.4	90.3 ± 22.8	91.7 ± 21.9
	PR	92 ± 25.9	90.8 ± 23.5	91.7 ± 24.6	91.8 ± 24.6
	TR	96.4 ± 24.3	93.3 ± 22.3	93.6 ± 23.1	94.5 ± 23.6
EtCO_2_ (mmHg)	NR	32.2 ± 6.2	31.7 ± 5.6	31.6 ± 5.7	31.7 ± 5.8
	PR	29.9 ± 5.6	29.8 ± 5.4	30.3 ± 5.6	29.9 ± 5.4
	TR	31 ± 6.6	31.9 ± 6.6	32.1 ± 7	32 ± 7.4
VTI (cm)	NR	18.2 ± 5.3	19 ± 5.8	18.8 ± 5.9	18.6 ± 5.9
	PR	14.3 ± 3.6	19.5 ± 4.9	18.6 ± 5.2	18.9 ± 5.3
	TR	16.2 ± 4.9	20.4 ± 5.5	18.1 ± 5.4	17.3 ± 5.4
E wave (m s^−1^)	NR	0.7 ± 0.2	0.9 ± 0.2	0.8 ± 0.2	0.8 ± 0.2
	PR	0.6 ± 0.2	0.8 ± 0.2	0.7 ± 0.2	0.7 ± 0.2
	TR	0.7 ± 0.2	0.8 ± 0.2	0.7 ± 0.2	0.7 ± 0.2
A wave (m s^−1^)	NR	0.7 ± 0.2	0.8 ± 0.2	0.8 ± 0.2	0.8 ± 0.2
	PR	0.8 ± 0.2	0.8 ± 0.2	0.8 ± 0.2	0.8 ± 0.2
	TR	0.7 ± 0.3	0.8 ± 0.3	0.8 ± 0.2	0.8 ± 0.3
E/A ratio	NR	1.1 ± 0.4	1.2 ± 0.4	1.1 ± 0.4	1.1 ± 0.4
	PR	0.9 ± 0.4	1 ± 0.4	1 ± 0.5	1 ± 0.5
	TR	1.1 ± 0.8	1.2 ± 1.2	1 ± 0.3	0.9 ± 0.3
E′ wave (m s^−1^)	NR	0.1 ± 0.1	0.2 ± 0.2	0.2 ± 0.3	0.1 ± 0.2
	PR	0.2 ± 0.3	0.3 ± 0.4	0.3 ± 0.4	0.2 ± 0.4
	TR	0.2 ± 0.2	0.1 ± 0.1	0.1 ± 0.2	0.1 ± 0.2
E/E′ ratio	NR	7.9 ± 4	8 ± 4.2	7.8 ± 3.7	8.3 ± 3.9
	PR	5.7 ± 3.4	6.5 ± 3.6	6 ± 3.6	6.4 ± 3.4
	TR	6.6 ± 3.2	8 ± 3	7.7 ± 3.3	7.4 ± 3.5
ScVO_2_ (%)	NR	74 ± 12.1	74.4 ± 11.7	NA	74 ± 12.1
	PR	76.1 ± 11.3	76.4 ± 8.2	NA	76.1 ± 11.3
	TR	78.5 ± 8.5	79.1 ± 9.2	NA	78.5 ± 8.5
Arterial lactate (mmol L^−1^)	NR	2.7 ± 2.4	2.7 ± 2.8	NA	2.6 ± 2.8
	PR	3.4 ± 2.6	3.0 ± 2.0	NA	3.2 ± 2.1
	TR	2.6 ± 2.1	2.3 ± 1.9	NA	2.5 ± 2.1

Continuous data are presented as the mean ± SD

*NR* non-responders, *TR* transient responders, *PR* persistent responders, *MAP* mean arterial pressure, *SAP* systolic arterial pressure, *DAP* diastolic arterial pressure, *HR* heart rate, *EtCO2* end tidal CO_2_, *VTI* sub-aortic velocity time integral, *ScVO*_*2*_ central venous oxygen saturation

## Discussion

### Key findings

The present study assessing the short-term hemodynamic effects of FC in sepsis shows that half of the responders at the end of the FC are no longer responders 20 min later (Fig. 3). These findings led to describe three different profiles of fluid responsiveness: the NR for whom no fluid efficacy was observed over time, the PR exhibiting a positive and sustained response to FC over time, and the TR exhibiting initially a positive response to FC that was not maintained over time. The VTI course over time was significantly different between NR, PR, and TR (*p* < 0.001) (Fig. 4) as well as baseline VTI (18.2 ± 5.3 cm, 14.3 ± 3.6 cm, 16.2 ± 4.9 cm, respectively). We assume that VTI values at baseline (reflecting blood volume status) could help identifying transient and persistent fluid responders.

### Relationship with previous papers

The time course of volume expansion after a fluid challenge has been poorly described. In experimental model, Guyton et al. demonstrated in normovolemic anesthetized dogs that fluid infusion increased by two to three times CO and mean circulatory pressure [19]. In this experiment, these two parameters returned to baseline values within 90 to 120 min. This experiment shows that in normovolemic conditions and preserved systolic function, the physiological response of cardiovascular system to fluid infusion is a transient increase in CO. A recent study including 20 patients with circulatory shock showed that hemodynamic effects of crystalloid infusion no longer last after 60 min, even in patients that have been considered as responders immediately after fluid infusion [20]. In this study, the author’s main hypothesis was that crystalloids infusion was systematically associated with capillary leak and subsequent decrease in plasma volume expansion over time. Similarly, in a recent randomized trial involving 200 postoperative hypovolemic patients, the infusion of lactated Ringer’s solution significantly improved cardiac output at the end of infusion, but this effect totally disappeared at 120 min [21]. Therefore, such results suggest that the immediate response to fluid does not predict the persistence of fluid efficacy over time. This is a key issue as it is widely recommended to assess fluid responsiveness at the end of fluid infusion or functional manoeuver such as passive leg raising (PLR) test. It was previously demonstrated that the maximal hemodynamic effect of PLR is observed from 30 to 90 s after the onset of the test [22]. Analogous results were recently reported after a conventional fluid challenge (250 ml crystalloid infusion over 5 min) [23]. In this study involving 26 postoperative patients, the authors demonstrated that the maximal CO increase was observed 1 min after the end of fluid infusion [23]. Moreover, the effect of fluid infusion was dissipated in 10 min both in R and NR patients. Therefore, the available literature suggests that fluid efficacy is never prolonged.

Moreover, in the present study, we used crystalloids for FC according to international guidelines [1, 15]. Several studies suggest that in normo- or slightly hypovolemic patients, the volume efficacy of crystalloids is closed to 20–25% [24] after 30 min of fluid infusion, due to a temporal shift of fluid toward interstitial compartment. This can explain the drop of VTI over time observed in TR that, in our hypothesis, are likely to be normovolemic. This hypothesis is supported by the fact that, even non-significant, the E/E′ ratio is more elevated in NR and TR (7.9 and 6.6) as compared to PR (5.7) (Table 4).

Conversely to previous studies focusing on the time course of fluid effects, we observed that some patients exhibited a sustained response to fluid therapy that we called “persistent response to fluid therapy.” In these patients, the hemodynamic values did not return to baseline 30 min after the start of the infusion. In PR, we could hypothesize that the efficacy of fluid is probably increased because of a significant drop in blood volume leading to very low values of hydrostatic pressure. Hahn and coworkers have nicely demonstrated that the volume efficacy of crystalloids was directly affected by blood volume status [14, 24]. In healthy volunteers undergoing blood removal, the elimination rate of a Ringer’s solution from the blood compartment was 4-fold higher in volunteers with no blood removal as compared to the same volunteers experiencing a 900-ml blood withdrawal. This suggests that the fluid efficacy of crystalloids may be as high as 80 to 100% in case of absolute blood volume reduction [24–26]. As these results were mainly observed in a model of controlled hemorrhage in healthy volunteers, we cannot extrapolate to ICU patients, in whom a 80–100% fluid efficacy of crystalloids is unlikely to be observed.

It is unlikely that vasopressors can influence the type of fluid response, transient or persistent, in the present study as no difference was observed between TR and PR in terms of vasopressor doses.

Finally, in the present population with a majority of patients with normal LVEF (Table 2), the absolute VTI value at baseline is probably of particular importance for explaining the differences between TR, PR, and NR groups in terms of fluid responsiveness status. Figure 4 shows a significant difference at baseline VTI between the three groups. In the PR group, baseline VTI value is significantly lower (14 cm) than in TR and NR, suggesting that absolute VTI could help to detect PR.

### Implications of study findings

Our results highlight that, among fluid responders, a few proportion of them have a sustained response to fluid. These findings enhance to closely test fluid responsiveness before administering large amounts of fluid (500 ml) and to follow this response over time while looking at the efficacy of fluid infusion on organ dysfunction. Besides, fluid responsiveness should be assessed both at the end of fluid bolus and 30 min after the start in order to identify PR and TR. Further studies are necessary to identify if different fluid strategy should be applied in TR and PR.

### Study limitations

We have some limitations to declare:

- First, the present study does not report any outcome endpoints.
- Second, one could argue that MAP and ScvO_2_ were in normal ranges at baseline, suggesting adequate blood volume and cardiac output, and ruling out indication of fluid infusion. However, during sepsis, SvO_2_ can be elevated due to microcirculatory alterations and may not reflect adequate tissue oxygenation explaining that fluid challenge has been performed despite normal SvO2 value in the present study. Similarly, fluid challenge has also been performed despite a mean 74 mmHg MAP value at baseline as normal MAP does not rule out potential excess of norepinephrine associated with occult hypovolemia.

Third, it could be objected that performing fluid responsiveness tests (such as dynamic indices or PLR test) before administering a 500-mL fluid challenge would have been more suitable in order to limit undue fluid infusion. Despite such maneuvers are very informative, they are used in less than 15% of patients in large recent cohort studies [8, 9]. In this pragmatic trial, the aim was to describe the evolution of fluid responsiveness over time for current practices.

We decided to use TTE to assess the response to fluid challenge even though some authors do not consider TTE as a valuable tool to assess fluid responsiveness [27]. However, guidelines on hemodynamic monitoring and a recent report consider TTE as reliable as thermodilution to assess CO [1, 28]. The reported intra- and inter-observer variability for VTI (aortic or pulmonary) measurement is closed to 5 to 8% with an intraclass correlation coefficient of 0.94 [12, 29].

## Conclusion

This study shows that 51.3% of initial responders have a persistent response to fluid 30 min after the beginning of fluid infusion and only 41.3% have a transient response. These findings highlight that fluid responsiveness is time-dependent and that the issue of optimal timing needs to be addressed in future studies.

## Additional file

Additional file 1: Sub-aortic velocity time integral (VTI) individual values. a In persistent responders. b In non-responders. c In transient responders. (PDF 190 kb)
