# Supplementary figures and images for: Time course of fluid responsiveness in sepsis: the fluid challenge revisiting (FCREV) study
Source: Crit Care. 2019 May 16;23:179. doi: 10.1186/s13054-019-2448-z (PMC6524325; doi:10.1186/s13054-019-2448-z)

VTI (cm)

Persistent responders

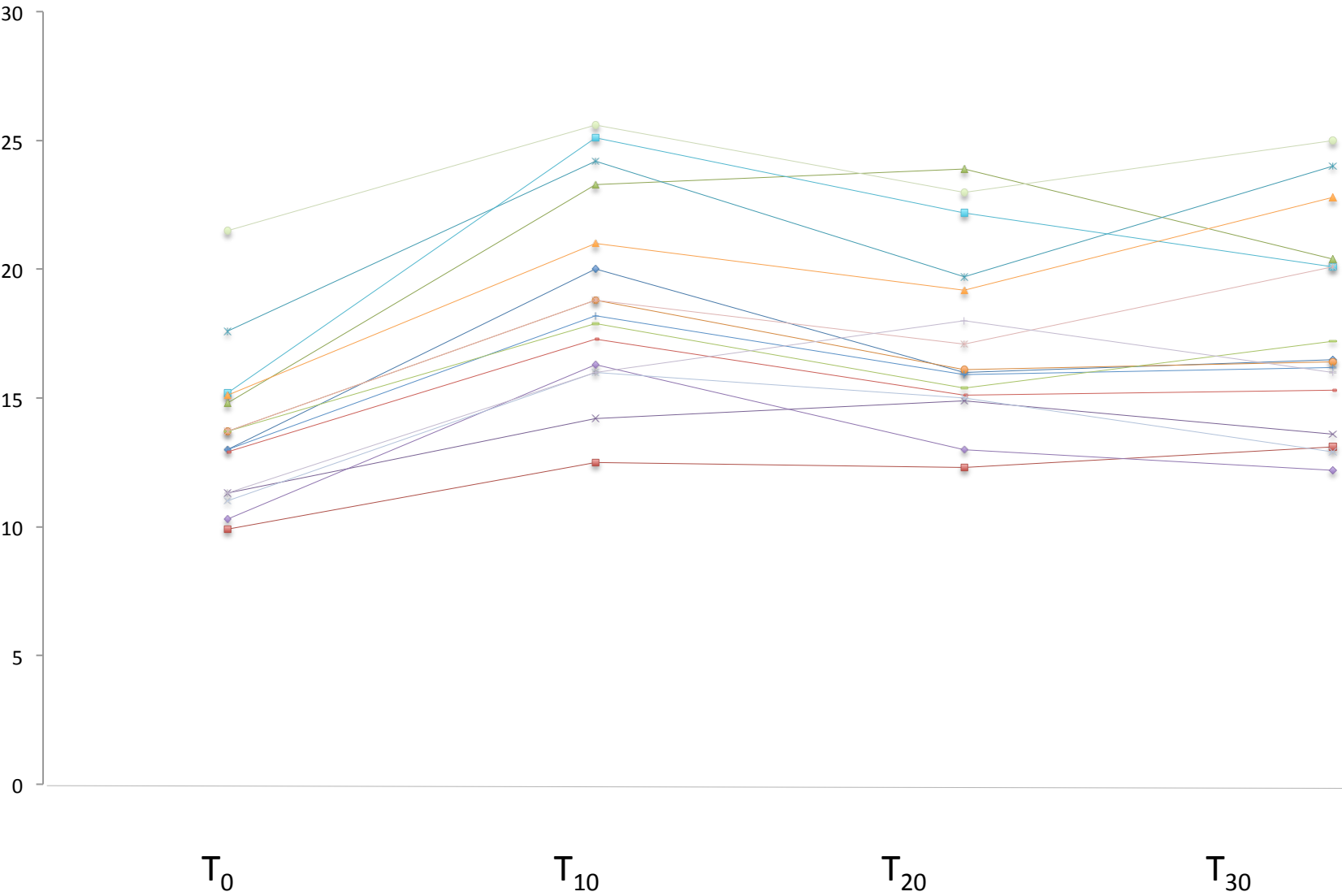

VTI (cm)

Non responders

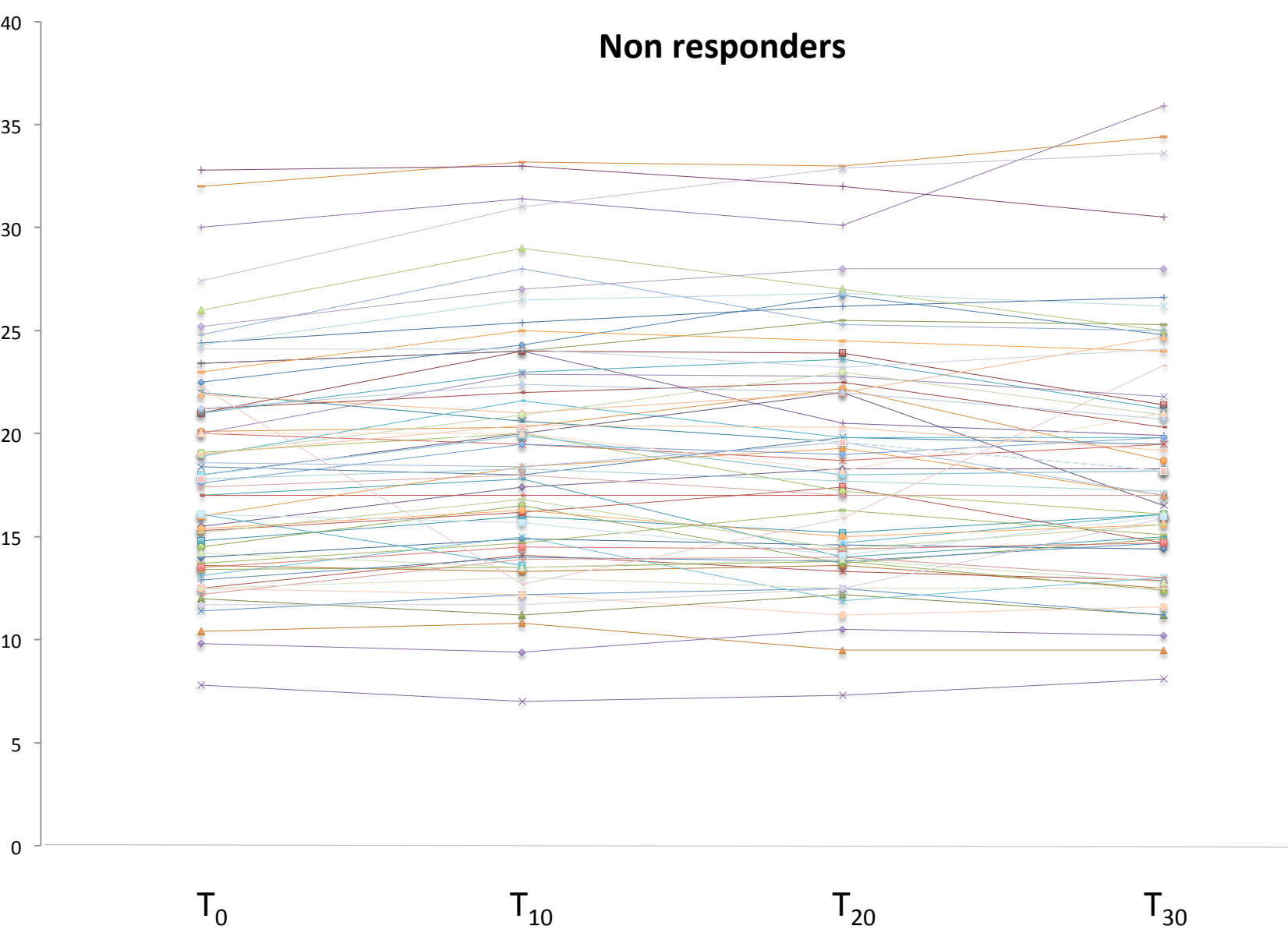

VTI (cm)

Transient responders

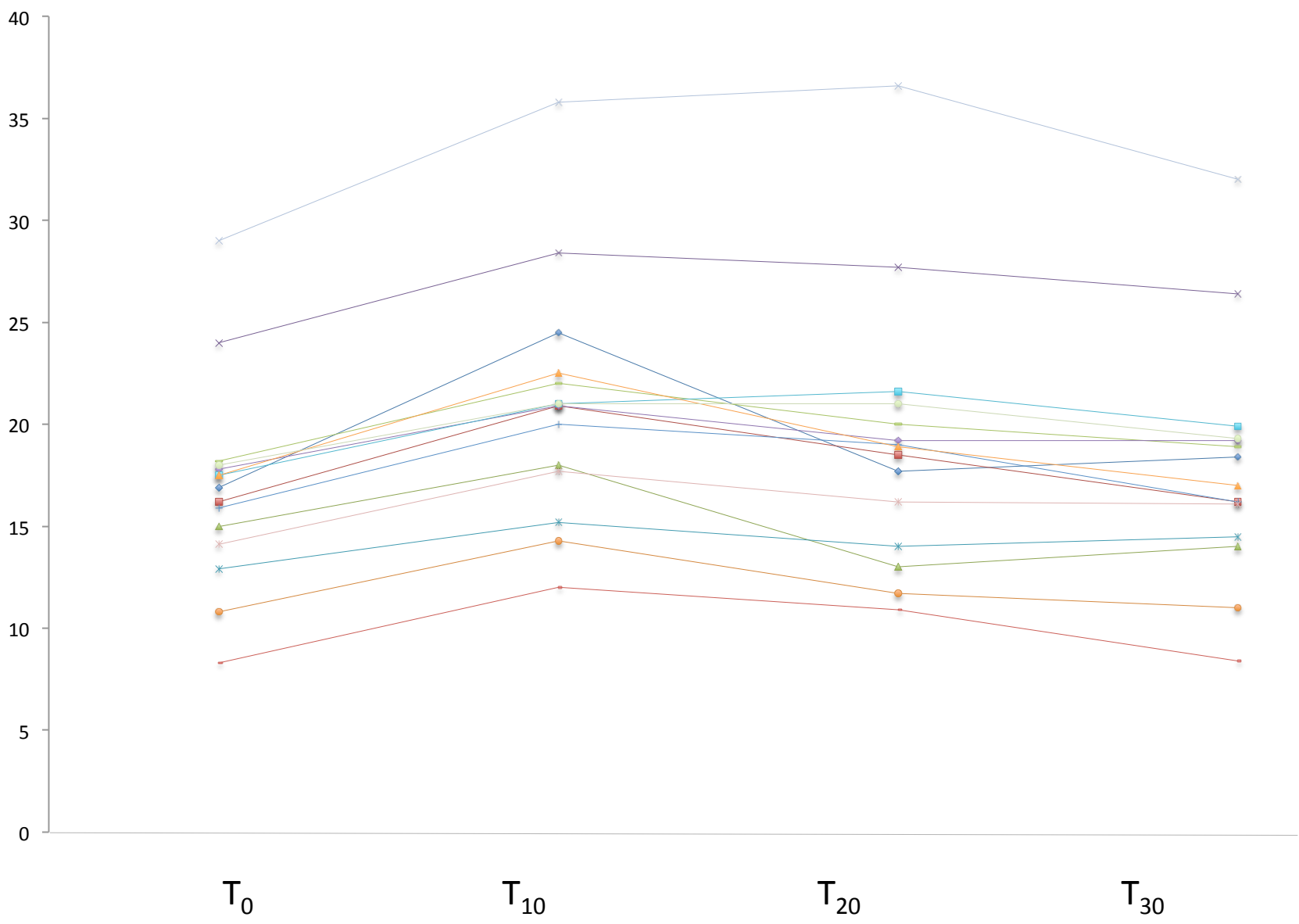

Supplement: Supplementary file 1 — Sub-aortic velocity time integral (VTI) individual values. a In persistent responders. b In non-responders. c In transient responders. (PDF 190 kb) [file 13054_2019_2448_MOESM1_ESM.pdf]
